# Case for diagnosis. Pregnant woman in the 3^rd^ trimester with pruritic papules and pustules on the trunk. Pruritic folliculitis of pregnancy^☆^

**DOI:** 10.1016/j.abd.2021.07.002

**Published:** 2021-11-20

**Authors:** Maria Rita Carvalho de Freitas Amorim, Flavia Amorim Meira Cavaliere, Esther Oliveira Xavier de Brito, Amanda Nascimento Cavalleiro de Macedo Mota

**Affiliations:** Service of Dermatology, Hospital Central da Aeronáutica, Rio de Janeiro, RJ, Brazil

## Case report

**Figure 1:**
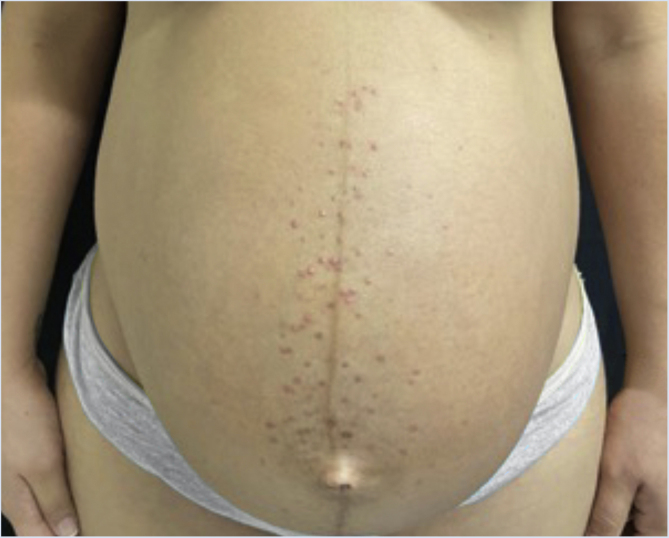
Erythematous papules and pustules located on the sternum and abdomen (*linea nigra*).

**Figure 2:**
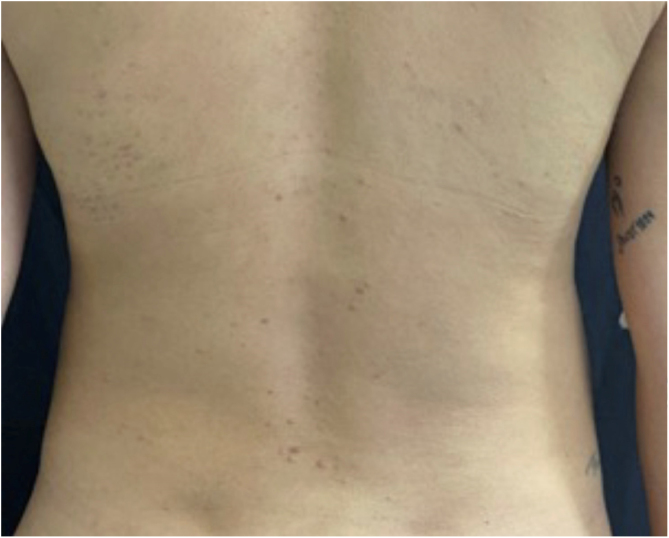
Erythematous papules located on the back.

**Figure 3:**
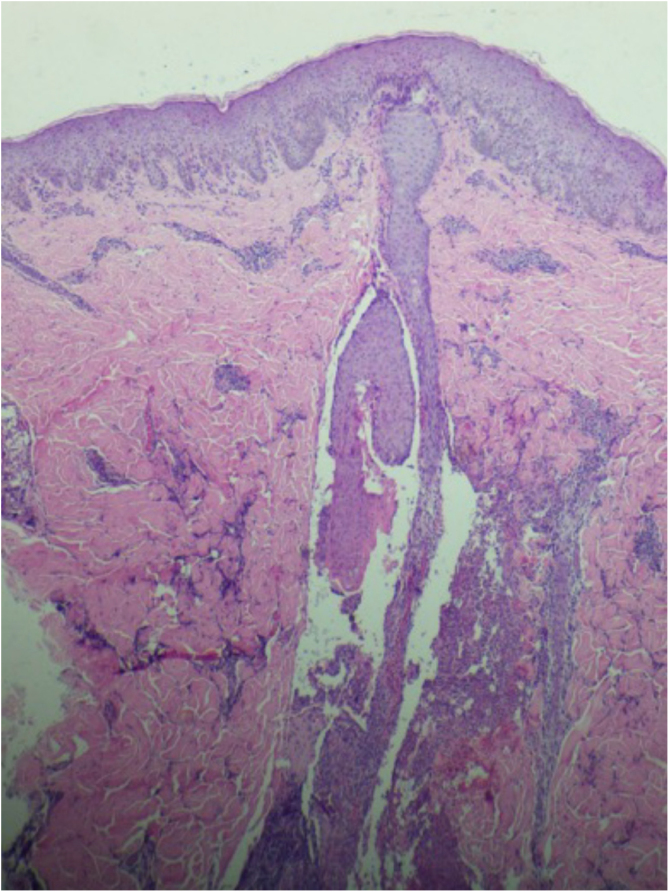
Perivascular infiltrate predominantly consisting of lymphocytes is observed in the dermis (Hematoxylin & eosin, ×40).

**Figure 4:**
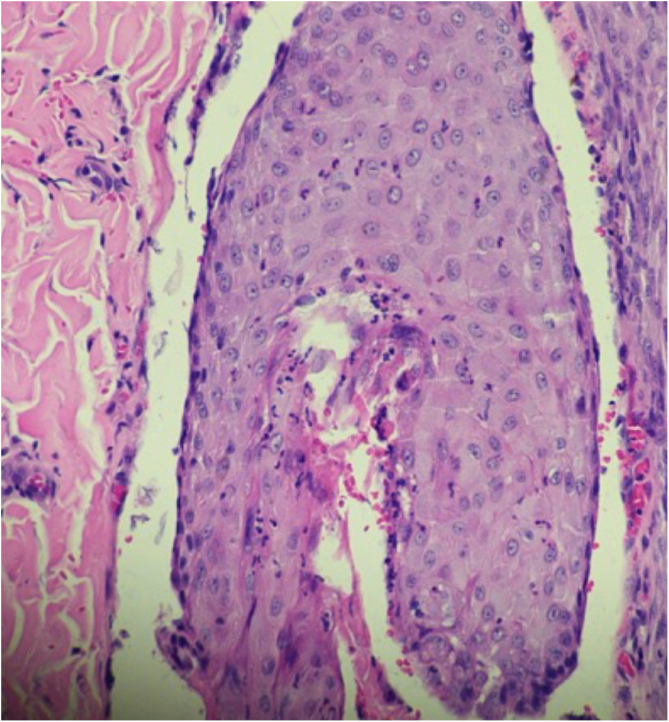
Hair follicle permeated by an inflammatory infiltrate consisting predominantly of neutrophils leading to the destruction of the follicular structure (Hematoxylin & eosin, ×400).

A 26-year-old primiparous female, at her 31^st^ week of gestation, reported the onset of pruritic skin lesions on the trunk, with two months of evolution. On physical examination, erythematous papules and pustules were observed on the abdomen (especially on the *linea nigra*), on the sternum, the back, and the lateral surface of the buttocks (Figure 1, Figure 2). Laboratory tests were requested and a biopsy of the dorsal pustule was performed. Laboratory tests did not disclose any abnormalities and the histopathological analysis showed a lymphocytic perivascular infiltrate, predominantly in the dermis, in addition to a hair follicle permeated by an inflammatory infiltrate with a predominance of neutrophils and destruction of the follicular structure (Figure 3, Figure 4). The patient was treated with 5% benzoyl peroxide gel, with complete regression of the lesions in the first week of postpartum.

## What is your diagnosis?

a) Pruritic folliculitis of pregnancy
b) Polymorphic eruption of pregnancy
c) Bacterial folliculitis
d) Acneiform eruption

## Discussion

Pruritic folliculitis of pregnancy (PFP) was originally described in 1981 by Zoberman and Farmer.^1^, ^2^, ^3^, ^4^ The authors reported an erythematous-papular, pruritic eruption in six pregnant women between the fourth and ninth months of pregnancy.^1^, ^2^, ^3^ Lesions spontaneously regressed within one month after delivery.^1^, ^2^, ^3^

This is the least common pregnancy-specific pruritic dermatosis.^2^ Since its description, few case reports have been published. The prospective study carried out by Roger et al. in 1994, which followed 3,192 pregnant women, identified 51 cases associated with pruritus (1.6%), with only one (0.03%) being associated with sterile follicular papules and pustules on the trunk, consistent with the diagnosis of PFP.^5^

In the 1999 study by Vaughan Jones et al., a cohort of 200 women with gestational dermatoses (GD) with a two-year follow-up identified PFP in 14 patients (7%).^6^ It is noteworthy that, in this article, serum androgen levels were measured and showed no changes when compared to the control group, ruling out the hypothesis of an etiology related to the increase in the levels of these hormones.^6^

In 2006, Ambros-Rudolph et al. carried out a retrospective study by analyzing the medical records of 505 pregnant women. PFP was diagnosed in only one patient (0.2%), with a history of eczema in childhood and a family history of atopy in two first-degree relatives. The authors proposed a new classification of GD, introducing the term atopic eruption of pregnancy (AEP) which encompasses PFP, prurigo of pregnancy, and eczema of pregnancy.^7^ This term has been criticized because not all pregnant women with AEP have a history of atopy, similarly the patient in this report.

As described in the literature and observed in the present case, patients respond well to topical treatments based on 5% or 10% benzoyl peroxide and 1% hydrocortisone.^2^, ^8^, ^9^ Complete spontaneous regression of the lesions is common within weeks after delivery.^9^

Pruritic folliculitis of pregnancy is a rare, benign and self-limited gestational dermatosis that does not interfere with maternal-fetal morbidity and mortality. It is essential that pregnant women be adequately diagnosed and advised, and followed-up with specialized care.

## Financial support

None declared.

## Authors’ contributions

Maria Rita Carvalho de Freitas Amorim: Collection, analysis and interpretation of data; writing of the manuscript; obtaining, analysis and interpretation of data; critical review of the literature and approval of the final version of the manuscript.

Flavia Amorim Meira Cavaliere: Critical review of important intellectual content; effective participation in research orientation; intellectual participation in the propaedeutic and therapeutic conduct and approval of the final version of the manuscript.

Esther Oliveira Xavier de Brito: Critical review of important intellectual content and approval of the final version of the manuscript.

Amanda Nascimento Cavalleiro by M. Mota da Silva: Design and planning of the study; critical review of important intellectual content; intellectual participation in the propaedeutic and therapeutic conduct and approval of the final version of the manuscript.

## Conflicts of interest

None declared.
